# Effects of Ti_3_C_2_Tx MXene Addition to a Co Complex/Ionic Liquid-Based Electrolyte on the Photovoltaic Performance of Solar Cells

**DOI:** 10.3390/molecules29061340

**Published:** 2024-03-18

**Authors:** Ju Hee Gu, Dongho Park, Kyung-Hye Jung, Byung Chul Lee, Yoon Soo Han

**Affiliations:** Department of Advanced Materials and Chemical Engineering, Daegu Catholic University, Gyeongsan 38430, Republic of Korea; gjh3170@naver.com (J.H.G.); donggeulhonyang@naver.com (D.P.); khjung@cu.ac.kr (K.-H.J.); bclee@kbsc.ac.kr (B.C.L.)

**Keywords:** dye-sensitized solar cell, redox mediator, Ti_3_C_2_Tx MXene, hole conduction

## Abstract

Redox mediators comprising I^−^, Co^3+^, and Ti_3_C_2_Tx MXene were applied to dye-sensitized solar cells (DSCs). In the as-prepared DSCs (I-DSCs), wherein hole conduction occurred via the redox reaction of I^−^/I_3_^−^ ions, the power conversion efficiency (PCE) was not altered by the addition of Ti_3_C_2_Tx MXene. The I-DSCs were exposed to light to produce Co^2+^/Co^3+^-based cells (Co-DSCs), wherein the holes were transferred via the redox reaction of Co^2+^/Co^3+^ ions. A PCE of 9.01% was achieved in a Co-DSC with Ti_3_C_2_Tx MXene (Ti_3_C_2_Tx-Co-DSC), which indicated an improvement from the PCE of a bare Co-DSC without Ti_3_C_2_Tx MXene (7.27%). It was also found that the presence of Ti_3_C_2_Tx MXene in the redox mediator increased the hole collection, dye regeneration, and electron injection efficiencies of the Ti_3_C_2_Tx-Co-DSC, leading to an improvement in both the short-circuit current and the PCE when compared with those of the bare Co-DSC without MXene.

## 1. Introduction

Two-dimensional (2D) materials are typically crystalline solids consisting of a single layer of atoms. These materials are considered promising for various applications, which explains why they remain the focus of research. MXenes with a general formula of M_n+1_X_n_T_x_ (where n = 1–3; M denotes a transition metal; X is either carbon or nitrogen; and T_x_ indicates surface terminal groups such as −OH, −F, −Cl, and/or −O−) were created by selectively etching the “A” layers from layered MAX phases (M_n+1_AX_n_, where A is usually any element from among Cd, Al, Si, P, S, Ga, Ge, As, In, Sn, Tl, and Pb [groups 12–16]) and can be easily solution-processed in aqueous or polar organic solvents due to their hydroxyl- or oxygen-terminated surfaces [1,2,3,4]. Following the production of multilayered Ti_3_C_2_Tx MXene by etching the Al layers from the Ti_3_AlC_2_ MAX phase in 2011 at Drexel University [1], numerous related research results have been reported in the fields of energy storage, sensors, light-emitting diodes, electromagnetic shielding, and environmental applications [2,3,4]. In addition, MXenes have been extensively studied in relation to applications concerning solar cells, given their metallic conductivity, excellent charge carrier mobility, high optical transmittance, and tunable work function [5,6,7,8]. Among the various MXenes, Ti_3_C_2_T_x_ is the most commonly studied in terms of third-generation solar cells, such as dye-sensitized solar cells (DSCs) [9,10,11], perovskite solar cells [12,13,14], and polymer solar cells [15,16]. Di and Qin reported that a power conversion efficiency (PCE) of 8.08% was achieved in a DSC with TiN@Ni-Ti_3_C_2_Tx MXene film as a counter electrode, surpassing that of a cell with Pt-based counter electrode (7.59%) [9]. A comparative study of 2D-layer-structured Ti_3_C_2_Tx MXene and TiC particles was reported. The DSCs with a Ti_3_C_2_Tx-based counter electrode achieved a PCE of 9.57%, much higher than that of the counterpart device with a TiC particle-based one (7.37%) [10]. It was also reported that DSCs with a poly(3,4-ethylene dioxythiophene)(PEDOT)/Ti_3_C_2_Tx MXene composite-based counter electrode outperformed cells with PEDOT- or Ti_3_C_2_Tx MXene-based ones in PCE [11].

A conventional DSC is composed of a dye-adsorbed TiO_2_ layer on a transparent electrode (i.e., a working electrode), a liquid electrolyte, and a Pt catalytic layer on a conductive electrode (i.e., a Pt counter electrode). Light absorption in dye molecules leads to the formation of excitons (electron–hole pairs), and the excited electrons are injected into the TiO_2_ layer. The photoinjected electrons and the holes in the dye molecules are transported to the electrodes via the TiO_2_ layer and the electrolyte, respectively. Finally, the electrons and holes are collected in the electrodes, allowing electron flows through the external circuits to occur [17,18]. The hole-conducting electrolyte comprises redox couples and electrical additives [19,20]. The redox couples, such as I^−^/I_3_^−^, Co^+2^/Co^+3^, Cu^+1^/Cu^+2^, and Ni^+3^/Ni^+4^, are reduced near the Pt counter electrode and oxidized near the excited dye molecules, thereby allowing for hole collection and dye regeneration, respectively. Electrical additives such as 4-tert-butylpyridine (TBP) and cations (lithium [Li^+^] or guanidinium [C(NH_2_)^3+^]) represent another important ingredient in a liquid electrolyte for enhancing the photovoltaic parameters of cells. These additives can control the potential of the redox couple, the surface state of the TiO_2_ semiconductor, the shift in the conduction band edge, and the interfacial charge recombination through being incorporating in small amounts [20]. Ti_3_C_2_T_x_ MXene was introduced as an additive for electrolytes to improve the photovoltaic performance of quasi-solid-state DSCs [21,22]. Sun et al. reported that, via the addition of Ti_3_C_2_T_x_ MXene to a quasi-solid-state electrolyte composed of an I^−^/I_3_^−^ redox couple and a melamine-formaldehyde (MF) sponge, the average PCE of the DSCs under a room light condition (1000 lux) was improved by 26.92% from that of the reference cell without MXene (23.35%) [21]. It was also reported that a PCE of 29.94% under a condition of 1000 lux was achieved through the incorporation of both reduced graphene oxide (rGO) and Ti_3_C_2_T_x_ to a quasi-solid-state electrolyte containing an I^−^/I_3_^−^ redox couple, polyethylene oxide (PEO), and poly(vinylidene fluoride-co-hexafluoropropylene) (PVDF-HFP) [22].

In this study, we report the effects of Ti_3_C_2_T_x_ MXene addition to a liquid electrolyte on the photovoltaic performance of cells. Ti_3_C_2_T_x_-dispersed liquid electrolytes based on a metal complex (tris(2-(1H-pyrazol-1-yl)-4-tert-butylpyridine)cobalt(III) tri[bis(trifluoromethane)sulfonimide] [FK209]) as a source of Co^3+^ and an ionic liquid (1-methyl-3-propylimidazolium iodine [MPII]) as a source of I^−^ (iodide) were first prepared. Then, DSCs with Ti_3_C_2_T_x_-dispersed Co^3+^/I^−^ liquid electrolytes (redox mediators) were fabricated and their photovoltaic properties were compared with those of the reference cell without Ti_3_C_2_T_x_ MXene. To the best of our knowledge, this is the first report on the effects of Ti_3_C_2_T_x_ addition to Co complex (Co^3+^)/ionic liquid (I^−^)-based redox mediators. The reported photovoltaic parameters (short-circuit current [*J_sc_*], open-circuit voltage [*V_oc_*], and fill factor [*FF*]) of the DSCs with Ti_3_C_2_T_x_ MXene are summarized in Table 1, including those of our devices with FK209 (Co^3+^)/MPII (I^−^)/Ti_3_C_2_T_x_-based liquid electrolytes.

## 2. Results and Discussion

### 2.1. Photovoltaic Performance of DSCs Based on Ti_3_C_2_T_x_-Incorporated Co^3+^/I^−^ Redox Mediators

In a previous report, we revealed that, through the simple mixing of MPII and FK209, triiodide (I_3_^−^) and Co^2+^(FK209) were produced via a chemical reaction (1) between the iodide (I^−^) of MPII and Co^3+^(FK209), where Co^2+^(FK209) and Co^3+^(FK209) are the Co^2+^ and Co^3+^ ions of FK209, respectively. Since the Co^3+^(FK209) was almost fully converted into Co^2+^(FK209), the redox mediators contained both I_3_^−^ and Co^2+^(FK209) as well as I^−^ (originating from non-reacted MPII) [23]. Therefore, hole conduction occurs through reactions (2)–(5) when as-prepared DSCs are exposed to the AM 1.5 condition, indicating that the I^−^/I_3_^−^ redox couple is involved in the dye regeneration (chemical Equation (4)) and hole collection (chemical Equation (5)). Here, we code the as-prepared DSCs based on the I^−^/I_3_^−^ redox couple as I-DSCs.

3I^−^ + 2Co^3+^(FK209) → I_3_^−^ + 2Co^2+^(FK209) (1)

2Dye + *hν* → 2Dye* (2)

2Dye* → 2Dye^+^ + 2e^−^ (TiO_2_) (3)

3I^−^ + 2Dye^+^ → I_3_^−^ + 2Dye (4)

I_3_^−^ + 2e^−^ (Pt) → 3I^−^ (5)

**Figure 1:**
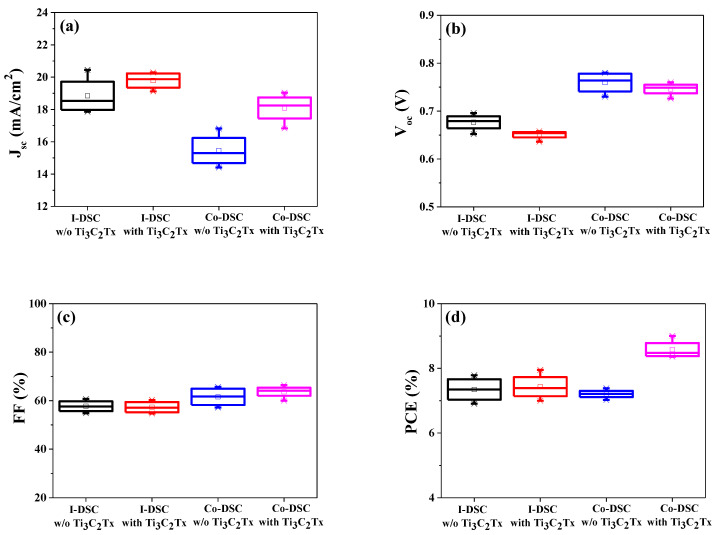
Performance comparison of the I- and Co-DSCs with or without Ti_3_C_2_T_x_ MXene: (**a**) *J_sc_*, (**b**) *V_oc_*, (**c**) *FF*, and (**d**) PCE measured under AM 1.5 irradiation.

Using the FK209 (Co^3+^)/MPII (I^−^) redox mediators with or without Ti_3_C_2_T_x_ MXene, DSCs were fabricated and the variations in the photovoltaic parameters were investigated. The average photovoltaic performance measured using the four I-DSCs (i.e., the as-prepared DSCs) is compared in Figure 1 and Table 2, while the raw data are presented in Table S1 in the Electronic Supplementary Materials (ESM). Through the incorporation of Ti_3_C_2_T_x_ MXene into the Co^3+^/I^−^ liquid redox mediators, the average *J_sc_* value of the I-DSCs was enhanced, whereas the average *V_oc_* value was decreased when compared with the values of the device without MXene, as shown in Figure 1a,b, respectively. As a result, the average PCE of the I-DSCs with Ti_3_C_2_T_x_ was very similar to that of the cells without Ti_3_C_2_T_x_.

Moreover, it was also found that chemical reactions (6) and (7) occurred through the exposure of the I-DSCs to light for a certain time [23]. Under illumination, the Co^2+^(FK209) that was produced via reaction (1) reduced the oxidized dye (Dye^+^), thereby regenerating Co^3+^(FK209) at the dye/redox mediator interface (chemical Equation (6)). The resulting Co^3+^(FK209) diffused to the counter electrode and then reduced to Co^2+^(FK209) through receiving an electron from the platinized FTO electrode (chemical Equation (7)), indicating that the Co^2+^/Co^3+^ redox couple is related to the dye regeneration (chemical Equation (6)) and hole collection (chemical Equation (7)). This may lead to an increase in the *V_oc_* value of the Co^2+^/Co^3+^-based cell when compared with that of the I^−^/I_3_^−^-based cell, as the potential gap between the conduction band edge (CBE) of the TiO_2_ and the redox potential (1.06 V versus a normal hydrogen electrode [NHE]) of the Co^2+^/Co^3+^(FK209) is wider than that (0.35 V versus a NHE) of the I^−^/I_3_^−^ electrolyte [24,25,26]. Here, we code the DSCs based on the Co^2+^/Co^3+^ redox couple as Co-DSCs, which were transformed from the I-DSCs via exposure to AM 1.5 light. To determine the time taken to convert the I-DSCs into Co-DSCs, we measured the *V_oc_* variations with the light exposure time of the I-DSCs. As shown in Figure S1, the *V_oc_* values improved with an increasing exposure time and then saturated after over a period of 150 min. This indicates that hole conduction mainly occurred through the action of the Co^2+^/Co^3+^ redox couple after exposure of the I-DSCs to light for over 150 min. To investigate effects of MXene incorporation in Co-DSCs, the I-DSCs with or without Ti_3_C_2_T_x_ MXene were exposed to the AM 1.5 condition for 150 min and their photovoltaic performance was measured. As a reference, the *V_oc_* values of the Co-DSCs were sharply increased when compared with those of the I-DSCs due to the wider potential gap between the TiO_2_’s CBE and the Co^2+^/Co^3+^(FK209)’s redox potential, as mentioned above [Figure 1b]. This suggests that the Co^2+^/Co^3+^(FK209) rather than the I^−^/I_3_^−^ redox couple participates in the hole conduction (chemical Equations (6) and (7)) in the Co-DSCs.

2Co^2+^(FK209) + 2Dye^+^ → 2Co^3+^(FK209) + 2Dye (6)

2Co^3+^(FK209) + 2e^−^ (Pt) → 2Co^2+^(FK209) (7)

**Figure 2:**
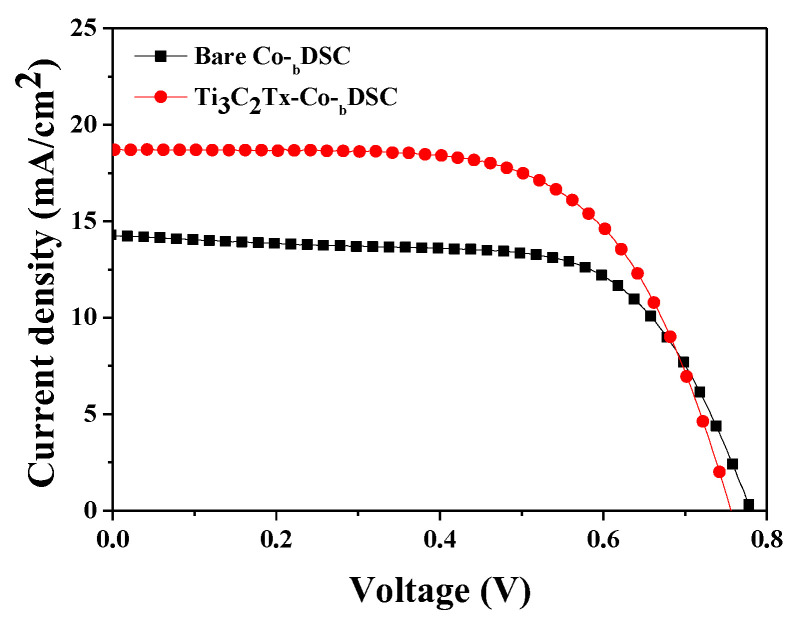
*J–V* characteristics of the best-performing cells—that is, the bare Co-_b_DSC and Ti_3_C_2_T_x_-Co-_b_DSC.

Through the addition of MXene, a substantial enhancement in the *J_sc_* and a slight decrement in the *V_oc_* values were observed in the Co-DSCs with Ti_3_C_2_T_x_ when compared with those of the reference cell without MXene, as shown in Figure 1a,b, respectively. There was no meaningful variation in the *FF* value, as demonstrated in Figure 1c. As a consequence, an improvement in the PCE was recorded in the MXene-incorporated Co-DSCs because the increase in the *J_sc_* overcame the decrement in the *V_oc_* value, as shown in Figure 1d. Among the four cells, we focused on the best-performing cells to reveal the origins of the improvement in the PCE via the incorporation of Ti_3_C_2_T_x_ MXene into Co^3+^/I^−^ liquid redox mediators. Here, we denote the best-performing Co-DSCs with or without Ti_3_C_2_T_x_ MXene as Ti_3_C_2_T_x_-Co-_b_DSC or bare Co-_b_DSC, respectively. Figure 2 presents the current density–voltage (*J–V*) curves of the Ti_3_C_2_T_x_- and bare Co-_b_DSCs, while the cell performance is compared in Table 3.

### 2.2. Effects of Ti_3_C_2_T_x_ Incorporation on the J_sc_ of Co-DSCs

**Figure 3:**
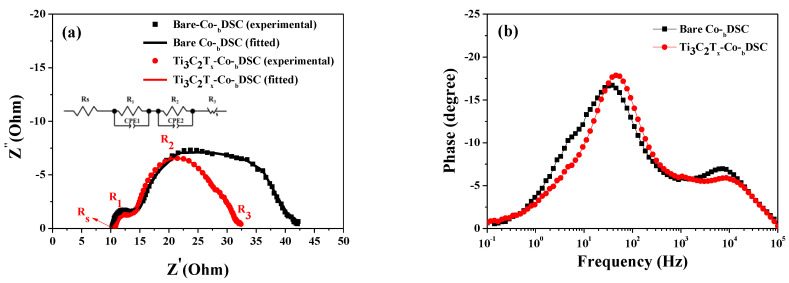
Nyquist (**a**) and Bode (**b**) plots of the EIS spectra for the bare and Ti_3_C_2_T_x_-Co-_b_DSCs, as measured under open-circuit conditions under the illumination of simulated AM 1.5 solar light.

Through the incorporation of Ti_3_C_2_T_x_ MXene into the Co^3+^/I^−^ redox mediator, the *J_sc_* value (18.45 mA/cm^2^) of the Ti_3_C_2_T_x_-Co-_b_DSC was substantially improved from that of the bare Co-_b_DSC (14.41 mA/cm^2^). It is believed that the conductive Ti_3_C_2_T_x_ MXene present in the Co^2+^/Co^3+^(FK209) electrolyte reduced the charge transfer resistance and improved the electrocatalytic performance between the Co^3+^(FK209) and the platinized FTO electrode, thereby leading to a reduction in the internal resistances and, therefore, an increase in the *J_sc_* value of the Ti_3_C_2_T_x_-Co-_b_DSC [21,22,27]. To confirm this, impedance spectroscopic (EIS) analysis was performed for the bare and Ti_3_C_2_T_x_-Co-_b_DSCs. Figure 3a shows the Nyquist plots of the EIS spectra of the Co-DSCs, as measured at the open-circuit condition under AM 1.5 one-sun illumination, providing the series (R_s_) and internal resistances. Three typical arcs, corresponding to the resistance (R_1_) of the redox reaction at the platinized FTO/electrolyte interface in the high-frequency region, the electron transfer resistance (R_2_) at the TiO_2_/dye/electrolyte interface in the medium-frequency region, and the ionic diffusion resistance (R_3_) within the electrolyte, were observed. The fitted resistances are compared in Table 4. A smaller R_1_ value (3.32 Ω) was measured in the Ti_3_C_2_T_x_-Co-_b_DSC when compared with that of the bare Co-_b_DSC (4.28 Ω). This indicates that the incorporation of Ti_3_C_2_T_x_ MXene can lead to an improvement in the electrocatalytic performance, causing an increase in the hole collection efficiency, when considering that the R_1_ value is related to the reduction of the Co^3+^(FK209) by the Pt catalyst (chemical Equation (7)) [21,22,27]. In addition, it is considered that the reduced resistance (R_1_) associated with chemical reaction (7) can effectively produce Co^2+^(FK209), resulting in the promotion of dye regeneration (chemical Equation (6)) [27], and thereby lowering the R_2_ value. The R_3_ values, named as Warburg diffusion resistance (Ws) arising from the ionic transport in the redox mediator [28], were almost similar in both the bare (6.79 Ω) and Ti_3_C_2_T_x_-Co-_b_DSCs (6.24 Ω), indicating that the presence of Ti_3_C_2_Tx MXene in the redox mediator did not hinder the diffusion of the Co^2+^/Co^3+^ redox couple.

**Figure 4:**
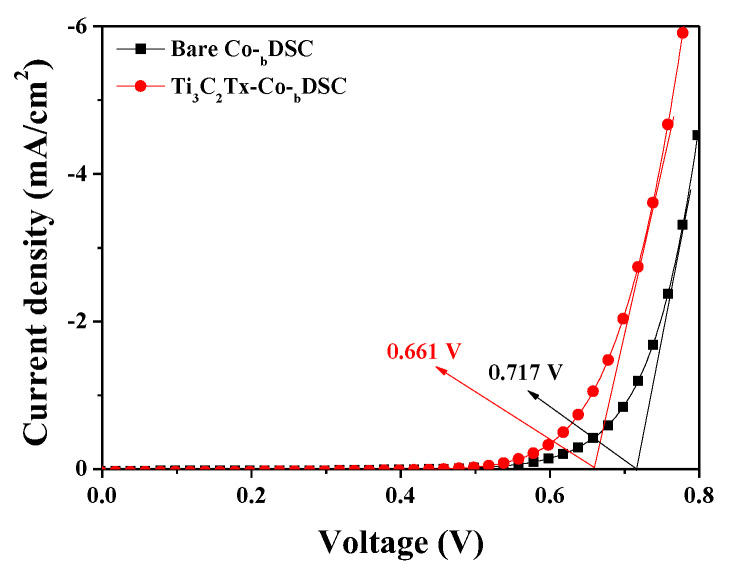
Dark current curves of the best-performing cells—that is, bare Co-_b_DSC and Ti_3_C_2_T_x_-Co-_b_DSC.

**Figure 5:**
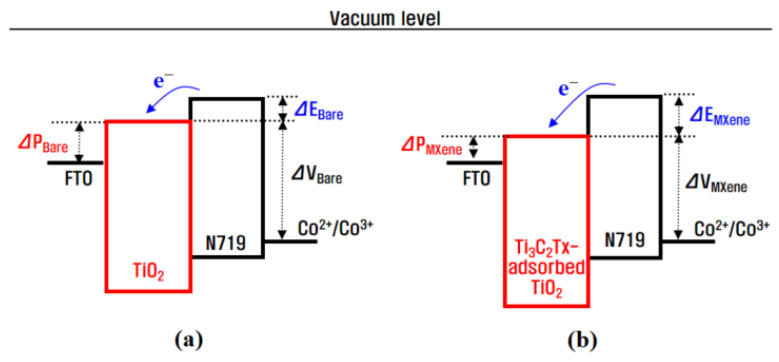
Schematic energy band diagram for the bare Co-_b_DSC (**a**) and Ti_3_C_2_T_x_-Co-_b_DSC (**b**), showing variations in the energy differences (ΔP_Bare_ > ΔP_MXene_, ΔE_Bare_ < ΔE_MXene_ and ΔV_Bare_ > ΔV_MXene_) by the positive shift in the TiO_2_’s CBE.

Furthermore, a shift in the TiO_2_’s CBE could be estimated from the dark current curves of the DSCs [29,30,31]. Figure 4 presents the dark current–voltage curves of the bare and Ti_3_C_2_T_x_-Co-_b_DSCs. The onset potential of the dark current for the bare Co-_b_DSC was estimated to be approximately 0.717 V, whereas the onset potential for the Ti_3_C_2_T_x_-Co-_b_DSC was shifted to approximately 0.661 V. Through the incorporation of Ti_3_C_2_T_x_ MXene, a lower onset potential was recorded, indicating that the potential difference (ΔP_MXene_) between the work function of the FTO and the TiO_2_’s CBE in the Ti_3_C_2_T_x_-Co-_b_DSC was smaller than that (ΔP_Bare_) in the bare Co-_b_DSC (i.e., ΔP_Bare_ > ΔP_MXene_) as illustrated in Figure 5. This suggests that the TiO_2_’s CBE in the Ti_3_C_2_T_x_-Co-_b_DSC was located at a more positive potential than that in the bare Co-_b_DSC. It is thought that the Ti_3_C_2_T_x_ MXene particles are adsorbed on the TiO_2_ surface, causing a surface dipole to be formed and resulting in a positive shift in the TiO_2_’s CBE. This positive shift (away from the vacuum level) in the CBE in the Ti_3_C_2_T_x_-Co-_b_DSC may lead to the more favorable injection of photoexcited electrons from the dye into the TiO_2_. It is because the potential difference (ΔE_MXene_) between the dye’s lowest unoccupied molecular orbital level (LUMO) and the TiO_2_’s CBE in the Ti_3_C_2_T_x_-Co-_b_DSC was larger than that (ΔE_Bare_) in the bare Co-_b_DSC (i.e, ΔE_Bare_ < ΔE_MXene_ in Figure 5), thereby resulting in an improvement in the electron injection efficiency [32]. Thus, it is considered that the positive shift in the TiO_2_’s CBE in the Ti_3_C_2_T_x_-Co-_b_DSC yielded a higher electron injection efficiency than the bare Co-_b_DSC. A similar result was reported in relation to the incorporation of Ti_3_C_2_T_x_ MXene into the mesoporous TiO_2_ layer, which induced a positive shift in the TiO_2_’s CBE, leading to an enhancement of the electron injection efficiency [33].

Meanwhile, the light scattered by the Ti_3_C_2_T_x_ MXene particles can affect the light-harvesting efficiency of cells, and thereby the *J_sc_* value. To confirm this, UV-visible absorption spectra of Co^3+^/I^−^ redox mediators with or without Ti_3_C_2_T_x_ MXene were measured, and compared with the absorption spectrum of N719 dye. As displayed in Figure S2 of the ESM, the Co^3+^/I^−^/Ti_3_C_2_T_x_ redox mediator showed strong and weak absorption at 200–500 nm and 500–1100 nm, respectively, indicating that incident light of 500–1100 nm can be in part scattered by the MXene particles. When considering that the absorption range of the N719 dye is 200–700 nm, the scattered light of 500–700 nm can be absorbed by the N719 dye and thus increase the light-harvesting efficiency. However, it was considered that the degree of increase in the light harvesting efficiency was not high, because the light of 500–700 nm was partly absorbed by the MXene particles. Actually, as shown in Figure S3 of the ESM, the deep brown-colored Co^3+^/I^−^/Ti_3_C_2_T_x_ redox mediator was due to the strong absorption at 200–500 nm and weak absorption at 500–700 nm. Overall, we attributed the enhanced *J_sc_* value in the Ti_3_C_2_T_x_-Co-_b_DSC to the increases in both the hole collection and the dye regeneration efficiency, which resulted from the reduced internal resistances, as well as to the improvement in the electron injection efficiency due to the positive shift in the TiO_2_’s CBE.

### 2.3. Effects of Ti_3_C_2_T_x_ Incorporation on the V_oc_ of Co-DSCs

The *V_oc_* value (0.760 V) of the Ti_3_C_2_T_x_-Co-_b_DSC was decreased when compared with that of the bare Co-_b_DSC (0.780 V). As expressed in Equation (8), the *V_oc_* value of the DSCs under constant illumination can be expressed as a function of the quasi-Fermi level of the semiconductor (*E_Fn_*) with respect to the dark value (*E_F_*_0_), which equals the electrolyte redox energy (*E_F_*_0_ = *E_redox_*). Therefore, it can be written with the thermal energy (*k_B_T*; 4.11 × 10^−21^ J at 25 °C), Boltzmann constant (*k_B_*), absolute temperature (*T*), positive elementary charge (*e*; 1.602 × 10^−19^ C), concentration in the dark (*n*_0_), and free electron density of the TiO_2_ photoelectrode (*n*) [34,35,36]. Equation (8) indicates that the *V_oc_* is affected by *n*, which is closely related to the back electron transfer (BET) reaction that occurs between the photoinjected electrons and the ions in the electrolyte. As the BET reaction decreases the *n* value, suppression of the BET reaction is necessary to increase the *V_oc_*. The *n* value can be estimated by measuring the lifetime of the electrons photoinjected into the TiO_2_, where a longer electron lifetime can increase the *n* value and, therefore, the *V_oc_*. Figure 3b shows Bode phase plots of the EIS spectra of the bare and Ti_3_C_2_T_x_-Co-_b_DSCs. Using the peak frequencies (*f_max_*) of 38.2 Hz and 45.5 Hz obtained from the EIS Bode phase plots of the bare and Ti_3_C_2_T_x_-Co-_b_DSCs, respectively, the electron lifetime (τ_n_) was estimated using Equation (9) [30,37]. The calculated electron lifetimes were 4.16 ms and 3.50 ms for the bare and Ti_3_C_2_T_x_-Co-_b_DSCs, respectively. A shortened lifetime on the part of the electrons injected from the photoexcited dyes was observed for the Ti_3_C_2_T_x_-Co-_b_DSC relative to that of the control cell (bare Co-_b_DSC), indicating that the BET reaction (chemical Equation (10)) in the Ti_3_C_2_T_x_-Co-_b_DSC occurred faster than that in the bare Co-_b_DSC.

(8) $$V_{oc}=\frac{E_{Fn}-E_{F0}}{e}=\frac{k_{B}T}{e}\ln(\frac{n}{n_{0}})$$

(9) $$\tau_{n}=\frac{1}{2\pi f_{max}}$$

Co^3+^(FK209) + e^−^ (TiO_2_) → Co^2+^(FK209) (10)

**Figure 6:**
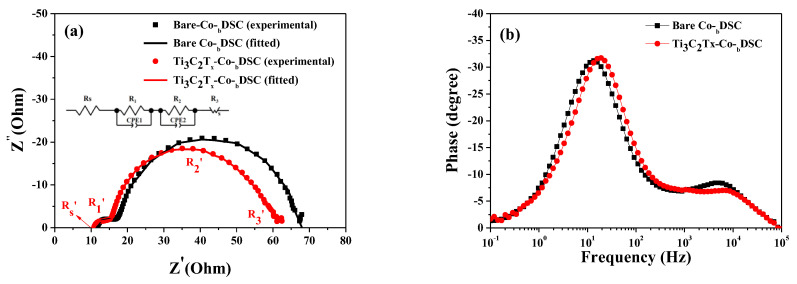
Nyquist (**a**) and Bode (**b**) plots of the EIS spectra for the bare- and Ti_3_C_2_T_x_-Co-_b_DSCs, as measured at −0.7 V in the dark.

To further confirm that the BET reaction was faster in the Ti_3_C_2_T_x_-Co-_b_DSC, Nyquist plots of the EIS spectra measured at −0.7 V in the dark were obtained, as shown in Figure 6a. When the EIS measurement is performed in the dark, electrons are injected from the FTO into the TiO_2_ under external applied voltage and then transferred to the electrolyte. Thus, the R_2_’ value of the Nyquist plot measured in the dark corresponds to the resistance of the BET reaction between the Co^3+^(FK209) in the electrolyte and the electrons injected into the TiO_2_ conduction band (chemical Equation (10)). It was observed that the arc of the impedance component R_2_’ (48.22 Ω) in the Ti_3_C_2_T_x_-Co-_b_DSC was smaller than that in the bare Co-_b_DSC (51.21 Ω). The smaller semicircle in the R_2_’ suggests that the BET reaction at the TiO_2_/dye/electrolyte interface was faster [38,39]. Moreover, from the peak frequencies (f_max_) given in Figure 6b and Equation (9), the lifetime of the electrons injected from the FTO was calculated to be 11.9 ms and 8.4 ms for the bare and Ti_3_C_2_T_x_-Co-_b_DSCs, respectively. The same tendency in terms of a shortened electron lifetime was observed in the measurements under illumination [Figure 3b] and dark conditions [Figure 6b].

**Figure 7:**
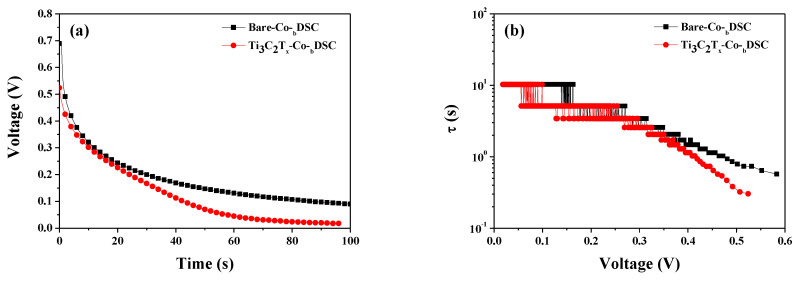
OCVD (**a**) and electron lifetime versus voltage (**b**) curves for the bare and Ti_3_C_2_T_x_-Co-_b_DSCs.

Another method to estimate electron lifetime in cells is open-circuit voltage decay (OCVD) measurements. Using the results of OCVD measurements [Figure 7a] and Equation (11), where *k_B_* is the Boltzmann constant, *T* is the temperature, *e* is the electron charge, and *dV_oc_*/*dt* is the derivative of the *V_oc_* transient [30,31,36], we could calculate the electron lifetime (τ) of the bare and Ti_3_C_2_T_x_-Co-_b_DSCs. As shown in Figure 7b, the electron lifetimes recorded for the Ti_3_C_2_T_x_-Co-_b_DSC were shorter than those for the reference cell (bare Co-_b_DSC), which suggests that the incorporation of the MXene boosted the BET reaction between the photoinjected electrons and the redox mediators. Overall, a faster BET reaction resulted in a lower *n* value, leading to a decrease in the *V_oc_* of the Ti_3_C_2_T_x_-Co-_b_DSC based on Equation (8).

(11) $$\tau =-\frac{k_{B}T}{e}{(\frac{dVoc}{dt})}^{-1}$$

Furthermore, the reduced *V_oc_* value in the Ti_3_C_2_T_x_-Co-_b_DSC can be explained by the positive shift in the TiO_2_’s CBE, as discussed above (Figure 4 and Figure 5). The lower *n* value can cause the positioning of the TiO_2_’s CBE to shift in a positive direction, lowering the *V_oc_* value. It is because the potential gap (ΔV_MXene_) between the TiO_2_’s CBE and the redox potential of the electrolyte in the Ti_3_C_2_T_x_-Co-_b_DSC was narrower than that (ΔV_Bare_) in the bare Co-_b_DSC (i.e., ΔV_Bare_ > ΔE_MXene_ in Figure 5) [40,41]. It is considered that this decrease in the *V_oc_* (or *n*) value originated from the adsorption of the Ti_3_C_2_T_x_ MXene on the TiO_2_ surface. More specifically, the electronically conductive Ti_3_C_2_T_x_ MXene particles could provide pathways for chemical reaction (10)—that is, the BET reaction between the photoinjected electrons and the Co^3+^(FK209) in the redox mediator. Meanwhile, it was known that pure Ti_3_C_2_ (or non-terminated Ti_3_C_2_) MXene had a metallic character, indicating its band gap energy (Eg) was zero (or Eg < 0.2 eV for the Ti_3_C_2_T_x_ MXene) [42,43]. It was also reported that the work function of Ti_3_C_2_T_x_ MXene varied from 3.9 to 4.8 eV with annealing temperature [44], which was positioned between the dye’s LUMO level (around 3.64 eV) and the TiO_2_’s CBE (around 5.11 eV). Thus, due to a lower position of the MXene’s work function than the dye’s LUMO level, another type of BET reaction between the photoexcited electrons in dyes and the Ti_3_C_2_T_x_ MXene can take place in the Ti_3_C_2_T_x_-Co-_b_DSC if the MXene particle comes into contact with the dye molecule. This can decrease the *n* value, and therefore the *V_oc_* value.

As a reference, the shorter electron lifetime observed in the Ti_3_C_2_T_x_-Co-_b_DSC may decrease the electron collection on the FTO and, therefore, the *J_sc_* value. In this study, it is believed that the enhanced hole collection, dye regeneration, and electron injection efficiencies surpassed the decreased electron collection efficiency.

### 2.4. Long-Term Stability of the Bare and Ti_3_C_2_T_x_-Co-_b_DSCs

**Figure 8:**
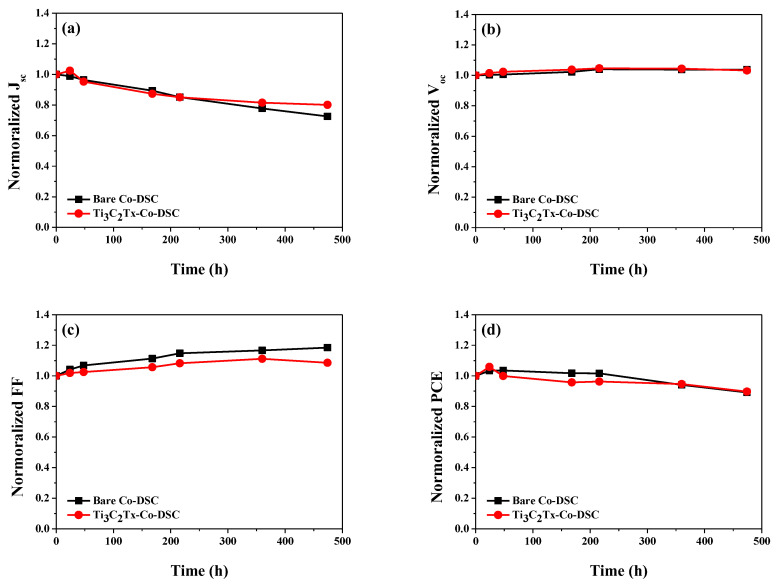
Variations in the photovoltaic performance over time: normalized *J_sc_* (**a**), *V_oc_* (**b**), *FF* (**c**), and PCE (**d**) of the bare and Ti_3_C_2_T_x_-Co-_b_DSCs stored at room temperature in the dark.

We compared the long-term stability of the bare and Ti_3_C_2_T_x_-Co-_b_DSCs by evaluating their photovoltaic properties over time. Here, the fabricated devices were additionally sealed using hot-melt glue sticks to minimize electrolyte leakage. Prior to the measurement of the photovoltaic performance, the I-DSCs with or without Ti_3_C_2_T_x_ MXene were converted into Co-DSCs through exposing them to the AM 1.5 condition for 150 min. Figure 8 compares the time-dependent performance variations in the cells stored at room temperature in the dark. Similar decay curves were noted in both devices, indicating that the incorporation of Ti_3_C_2_T_x_ mXene into the redox mediator did not affect the devices’ stability.

## 3. Materials and Methods

### 3.1. Materials

To fabricate DSCs, the same materials as those used in our previous report were utilized [23]. Their detailed information is provided in the ESM. The acetonitrile solvent used to prepare the liquid electrolytes was procured from Daejung Chemicals and Metals Co., Ltd. (Siheung, Republic of Korea). Colloidal suspension of single-layer Ti_3_C_2_ in acetonitrile (2 mg Ti_3_C_2_/mL) (BK2020082105-08) was purchased from Beijing Beike New Material Technology Co., Ltd. (Suzhou, China). All the chemicals used for DSC fabrication were exploited without further purification. The single-layer Ti_3_C_2_Tx MXene structure is illustrated in Figure S4 in the ESM. The chemical structures of the main components (FK209 and MPII) and additives (LiTFSI and TBP) of the electrolyte are shown in Figure S5 in the ESM.

### 3.2. Preparation of Co^3+^/I^−^-Based Liquid Electrolytes with or without Ti_3_C_2_T_x_

The Ti_3_C_2_T_x_-dispersed liquid electrolyte based on a Co^3+^/I^−^ redox mediator was prepared by dissolving MPII (0.6 M, 302.5 mg), FK209 (0.015 M, 15.2 mg), TBP (0.11 M, 32.4 mg), and LiTFSI (0.04 M, 22.8 mg) in the colloidal suspension of single-layer Ti_3_C_2_ in acetonitrile (2 mL). For the purpose of comparison, Co^3+^/I^−^-based liquid electrolytes without Ti_3_C_2_Tx were also prepared by simply replacing the Ti_3_C_2_Tx colloid with acetonitrile solvent (2 mL).

### 3.3. Fabrication of DSCs

Similar procedures to those described in our previous work were utilized to prepare the working (glass/FTO/TiO_2_:dye) and counter (glass/FTO/Pt) electrodes for the DSCs [23]. A 25 μm thick hot-melt adhesive was placed between the working and counter electrodes and then annealed for 10 min at 120 °C to seal the two electrodes. The Co^3+^/I^−^-based liquid electrolytes with or without Ti_3_C_2_T_x_ MXene were injected into the cells through one of the two small holes predrilled into the counter electrodes. By sealing the two holes, we were able to fabricate DSCs with a 25 mm^2^ active area. Detailed procedures are mentioned in the ESM.

### 3.4. Measurements

Photovoltaic performance measurements, EIS analyses, and dark current studies were carried out. UV-visible absorbance and OCVD measurements were also performed. All the measurements were performed under ambient conditions at room temperature. Detailed information for the measuring instruments is presented in the ESI.

## 4. Conclusions

The photovoltaic properties of DSCs with or without Ti_3_C_2_Tx MXene in Co^3+^/I^−^-based redox mediators were investigated in this study. Through the incorporation of Ti_3_C_2_T_x_ MXene into the Co^3+^/I^−^ liquid redox mediators, the average *J_sc_* value of the I-DSCs, in which hole conduction occurred via the redox reaction of the I^−^ and I_3_^−^ ions, was enhanced, whereas the average *V_oc_* value was decreased when compared with that of the device without the MXene. As a result, the average PCE of the I-DSCs with Ti_3_C_2_T_x_ was very similar to that of the cells without Ti_3_C_2_T_x_. To obtain Co-DSCs based on the Co^2+^/Co^3+^ redox couple, the I-DSCs were exposed to light for 150 min. Through the addition of Ti_3_C_2_Tx MXene into the Co^3+^/I^−^-based redox mediators, the hole collection, dye regeneration, and electron injection efficiencies of the Ti_3_C_2_Tx-Co-DSCs were all increased, leading to an improvement in both the *J_sc_* and PCE when compared with those of the bare Co-DSCs without MXene. These results indicate that Ti_3_C_2_Tx MXene, as a *J_sc_*-improver, is a good additive for improving the PCE of Co-DSCs.

## Figures and Tables

**Table 1:** Photovoltaic performances of DSCs with Ti_3_C_2_T_x_-MXene-incorporated electrolytes.

Device	Measurement Condition	Redox Mediator		*J_sc_* (mA/cm^2^)	*V_oc_* (V)	*FF* (%)	PCE (%)	Ref.
Quasi-solid-state DSC	AM 1.5	MF-sponge-based I^−^/I_3_^−^	Without Ti_3_C_2_Tx	14.979 ± 0.175	0.778 ± 0.004	65.3 ± 0.3	7.610 ± 0.106	[21]
			With Ti_3_C_2_Tx	15.085 ± 0.188	0.781 ± 0.003	66.4 ± 0.6	7.822 ± 0.092	[21]
	1000 lux	MF-sponge-based I^−^/I_3_^−^	Without Ti_3_C_2_Tx	0.177 ± 0.001	0.569 ± 0.007	70.3 ± 0.5	23.35 ± 0.43	[21]
			With Ti_3_C_2_Tx	0.196 ± 0.003	0.579 ± 0.004	71.9 ± 0.4	26.92 ± 0.43	[21]
	AM 1.5	PEO/PVDH-HFP-based I^−^/I_3_^−^	Without rGO/Ti_3_C_2_Tx	-	-	-	-	-
			With rGO/Ti_3_C_2_Tx	15.170 ± 0.203	0.783 ± 0.002	69.5 ± 0.5	8.255 ± 0.109	[22]
	1000 lux	PEO/PVDH-HFP-based I^−^/I_3_^−^	Without Ti_3_C_2_Tx	-	-	-	-	-
			With rGO	0.189 ± 0.002	0.544 ± 0.002	76.1 ± 0.4	23.22 ± 0.43	[22]
			With rGO/Ti_3_C_2_Tx	0.223 ± 0.001	0.561 ± 0.004	75.7 ± 0.1	29.94 ± 0.49	[22]
Liquid electrolyte DSC	AM 1.5	Co^3+^/ I^−^ (FK209/MPII)	Without Ti_3_C_2_Tx	15.46 ± 1.04	0.760 ± 0.023	61.33 ± 3.70	7.18 ± 0.11	This study
			With Ti_3_C_2_Tx	18.09 ± 0.94	0.746 ± 0.014	63.66 ± 2.69	8.58 ± 0.30	This study

**Table 2:** Averages and standard deviations of the cell performances measured using four I- and Co-DSCs with or without Ti_3_C_2_T_x_ MXene.

Devices		*J_sc_* (mA/cm^2^)	*V_oc_* (V)	*FF* (%)	PCE (%)
I-DSC	Without Ti_3_C_2_T_x_	18.84 ± 1.18	0.677 ± 0.018	57.70 ± 2.55	7.35 ± 0.39
	With Ti_3_C_2_T_x_	19.95 ± 0.78	0.651 ± 0.010	57.29 ± 2.59	7.43 ± 0.40
Co-DSC	Without Ti_3_C_2_T_x_	15.46 ± 1.04	0.760 ± 0.023	61.33 ± 3.70	7.18 ± 0.11
	With Ti_3_C_2_T_x_	18.09 ± 0.94	0.746 ± 0.014	63.66 ± 2.69	8.58 ± 0.30

**Table 3:** Photovoltaic performance of the best-performing cells—that is, the bare Co-_b_DSC and Ti_3_C_2_T_x_-Co-_b_DSC.

Best-Performing Cells	*J_sc_* (mA/cm^2^)	*V_oc_* (V)	*FF* (%)	PCE (%)
Bare Co-_b_DSC	14.41	0.780	64.66	7.27
Ti_3_C_2_T_x_-Co-_b_DSC	18.45	0.760	64.23	9.01

**Table 4:** Resistances for the Nyquist plots of the bare and Ti_3_C_2_T_x_-Co-_b_DSCs.

EIS Measurement Condition	Devices	R_s_ (Ω)	R_1_ (Ω)	R_2_ (Ω)	R_3_ (Ω)
Open-circuit	Bare Co-_b_DSC	10.24	4.28	30.88	6.79
	Ti_3_C_2_T_x_-Co-_b_DSC	10.77	3.32	22.02	6.24
Dark	Bare Co-_b_DSC	11.40	5.42	51.21	3.16
	Ti_3_C_2_T_x_-Co-_b_DSC	10.82	3.44	48.22	3.14

## Data Availability

Data are contained within the article and the Supplementary Materials.

## Supplementary Materials

The following supporting information can be downloaded at https://www.mdpi.com/article/10.3390/molecules29061340/s1: Table S1: Photovoltaic parameters of I- and Co-DSCs with or without Ti_3_C_2_T_x_ MXene; Figure S1: Improvement in *V_oc_* values by exposing I-DSCs to AM 1.5 light; Figure S2: UV-visible spectra of (a) Co^3+^/I^−^- and (b) Co^3+^/I^−^/Ti_3_C_2_Tx MXene-based redox mediators, and (c) N719 dye; Figure S3: Photographs of (a) Co^3+^/I^−^- and (b) Co^3+^/I^−^/Ti_3_C_2_Tx MXene-based redox mediators; Figure S4: Illustration of single-layered Ti_3_C_2_Tx MXene structure; Figure S5: Chemical structures of (a) FK209, (b) MPII, (c) LiTFSI, and (d) TBP.

## References

[1] Naguib M., Kurtoglu M., Presser V., Lu J., Niu J., Heon M., Hultman L., Gogotsi Y. (2011). Two-dimensional nanocrystals produced by exfoliation of Ti_3_AlC_2_. Adv. Mater..

[2] Papadopoulou K.A., Chroneos A., Parfitt D., Christopoulos S.-R.G. (2020). A perspective on MXenes: Their synthesis, properties, and recent applications. J. Appl. Phys..

[3] Jun B.-M., Kim S., Heo J., Park C.M., Her N., Jang M., Huang Y., Han J., Yoon Y. (2019). Review of MXenes as new nanomaterials for energy storage/delivery and selected environmental applications. Nano Res..

[4] Jimmy J., Kandasubramanian B. (2020). MXene functionalized polymer composites: Synthesis and applications. Eur. Polym. J..

[5] Saeed M.A., Shahzad A., Rasool K., Mateen F., Oh J.-M., Shim J.W. (2022). 2D MXene: A potential candidate for photovoltaic cells? a critical review. Adv. Sci..

[6] Yin L., Li Y., Yao X., Wang Y., Jia L., Liu Q., Li J., Li Y., He D. (2021). MXenes for solar cells. Nano-Micro Lett..

[7] Shi Z., Khaledialidusti R., Malaki M., Zhang H. (2021). MXene-based materials for solar cell applications. Nanomaterials.

[8] Qamar S., Fatima K., Ullah N., Akhter Z., Waseem A., Sultan M. (2022). Recent progress in use of MXene in perovskite solar cells: For interfacial modification, work-function tuning, and additive engineering. Nanoscale.

[9] Di Y., Qin T. (2022). Efficient wide-spectrum dye-sensitized solar cell by plasmonic TiN@Ni-MXene as electrocatalyst. Ceram. Int..

[10] Ma J.-Y., Sun M., Zhu Y.-A., Zhou H., Wu K., Xiao J., Wu M. (2020). Highly effective 2D layer structured titanium carbide electrode for dye-sensitized and perovskite solar cells. ChemElectroChem.

[11] Nagalingam S.P., Grace A.N. (2022). Poly(3,4-ethylenedioxythiophene) decorated MXene as an alternative counter electrode for dye-sensitized solar cells. Mater. Today Chem..

[12] Chen T., Tong G., Xu E., Li H., Li P., Zhu Z., Tang J., Qi Y., Jiang Y. (2019). Accelerating hole extraction by inserting 2D Ti_3_C_2_-MXene interlayer to all inorganic perovskite solar cells with long-term stability. J. Mater. Chem. A.

[13] Jin X., Yang L., Wang X.-F. (2021). Efficient two-dimensional perovskite solar cells realized by incorporation of Ti_3_C_2_T_x_ MXene as nano-dopants. Nano-Micro Lett..

[14] Agresti A., Pazniak A., Pescetelli S., Di Vito A., Rossi D., Pecchia A., Auf der Maur M., Liedl A., Larciprete R., Kuznetsov D.V. (2019). Titanium-carbide MXenes for work function and interface engineering in perovskite solar cells. Nat. Mater..

[15] Hou C., Yu H. (2020). Modifying the nanostructures of PEDOT:PSS/Ti_3_C_2_T_X_ composite hole transport layers for highly efficient polymer solar cells. J. Mater. Chem. C.

[16] Hou C., Yu H., Huang C. (2019). Solution-processable Ti_3_C_2_T_x_ nanosheets as an efficient hole transport layer for high-performance and stable polymer solar cells. J. Mater. Chem. C.

[17] Gong J., Sumathya K., Qiao Q., Zhou Z. (2017). Review on dye-sensitized solar cells (DSSCs): Advanced techniques and research trends. Renew. Sustain. Energy Rev..

[18] Sharma K., Sharma V., Sharma S.S. (2018). Dye-sensitized solar cells: Fundamentals and current status. Nanoscale Res. Lett..

[19] Wu J., Lan Z., Lin J., Huang M., Huang Y., Fan L., Luo G. (2015). Electrolytes in dye-sensitized solar cells. Chem. Rev..

[20] Iftikhar H., Sonai G.G., Hashmi S.G., Nogueira A.F., Lund P.D. (2019). Progress on electrolytes development in dye-sensitized solar cells. Materials.

[21] Wen J., Liu Y., Li T., Liu C., Wang T., Liu Y., Zhou Y., Li G., Sun Z. (2023). Low cost and strongly adsorbed melamine formaldehyde sponge electrolyte for nontraditional quasi-solid dye-sensitized solar cells. ACS Appl. Energy Mater..

[22] Wen J., Sun Z., Qiao Y., Zhou Y., Liu Y., Zhang Q., Liu Y., Jiao S. (2022). Ti_3_C_2_ MXene-reduced graphene oxide composite polymer-based printable electrolyte for quasi-solid-State dye-sensitized solar cells. ACS Appl. Energy Mater..

[23] Lee Y., Kwon Y., Cho Y., Ahn K.-S., Han Y.S. (2022). Novel heterologous binary redox mediator based on an ionic liquid and cobalt complex for efficient organic-solvent-free dye-sensitized solar cells. J. Ind. Eng. Chem..

[24] Noh J.H., Jeon N.J., Choi Y.C., Nazeeruddin M.K., Grätzel M., Seok S.I. (2013). Nanostructured TiO_2_/CH_3_NH_3_PbI_3_ heterojunction solar cells employing spiro-OMeTAD/Co-complex as hole-transporting material. J. Mater. Chem. A.

[25] Zhang Y., Sun Z., Shi C., Yan F. (2016). Highly efficient dye-sensitized solar cells based on low concentration organic thiolate/disulfide redox couples. RSC Adv..

[26] Xu D., Zhang H., Chen X., Yan F. (2013). Imidazolium functionalized cobalt tris(bipyridyl) complex redox shuttles for high efficiency ionic liquid electrolyte dye-sensitized solar cells. J. Mater. Chem. A.

[27] Chen X., Tang Q., He B., Lin L., Yu L. (2014). Platinum-free binary Co-Ni alloy counter electrodes for efficient dye-sensitized solar cells. Angew. Chem..

[28] Wang H., Liu M., Yan C., Bell J. (2012). Reduced electron recombination of dye-sensitized solar cells based on TiO_2_ spheres consisting of ultrathin nanosheets with [001] facet exposed. Beilstein J. Nanotechnol..

[29] Diamant Y., Chen S.G., Melamed O., Zaban A. (2003). Core-shell nanoporous electrode for dye sensitized solar cells: The effect of the SrTiO_3_ shell on the electronic properties of the TiO_2_ core. J. Phys. Chem. B.

[30] Baek G.W., Kim Y.-J., Jung K.-H., Han Y.S. (2019). Enhancement of solar cell performance through the formation of a surface dipole on polyacrylonitrile-treated TiO_2_ photoelectrodes. J. Ind. Eng. Chem..

[31] Shin S., Kim J., Kwon S.-J., Ryu K.H., Choi B., Han Y.S. (2023). Enhancement of photovoltaic performance of solvent-free dye-sensitized solar cells with doped poly(3-hexylthiophene). J. Ind. Eng. Chem..

[32] Watson D.F., Meyer G.J. (2004). Cation effects in nanocrystalline solar cells. Coord. Chem. Rev..

[33] Lemos H.G., Ronchi R.M., Portugal G.R., Rossato J.H.H., Selopal G.S., Barba D., Venancio E.C., Rosei F., Arantes J.T., Santos S.F. (2022). Efficient Ti_3_C_2_T_x_ MXene/TiO_2_ hybrid photoanodes for dye-sensitized solar cells. ACS Appl. Energy Mater..

[34] Peng T., Shi W., Wu S., Ying Z., Ri J.H. (2015). Sea urchin-like TiO_2_ microspheres as scattering layer of nanosized TiO_2_ film-based dye-sensitized solar cell with enhanced conversion efficiency. Mater. Chem. Phys..

[35] Zaban A., Greenshtein M., Bisquert J. (2003). Determination of the Electron lifetime in nanocrystalline dye solar cells by open-circuit voltage decay measurements. ChemPhysChem.

[36] Kim J.Y., Kim K.H., Kim D.-H., Han Y.S. (2020). Effects of a dianion compound as a surface modifier on the back reaction of photogenerated electrons in TiO_2_-based solar cells. Arab. J. Chem..

[37] Kim K.S., Song H., Nam S.H., Kim S.-M., Jeong H., Kim W.B., Jung G.Y. (2012). Fabrication of an efficient light-scattering functionalized photoanode using periodically aligned ZnO hemisphere crystals for dye-sensitized solar cells. Adv. Mater..

[38] Zhao J., Sun B., Qiu L., Cao H., Li Q., Chen X., Yan F. (2012). Efficient light-scattering functionalized TiO_2_ photoanodes modified with cyanobiphenyl-based benzimidazole for dye-sensitized solar cells with additive-free electrolytes. J. Mater. Chem..

[39] Tian H., Hu L., Zhang C., Liu W., Huang Y., Mo L., Guo L., Sheng J., Dai S. (2010). Retarded charge recombination in dye-sensitized nitrogen-doped TiO_2_ solar cells. J. Phys. Chem. C.

[40] Park K.-H., Dhayal M. (2009). High efficiency solar cell based on dye sensitized plasma treated nano-structured TiO_2_ films. Electrochem. Commun..

[41] Mandal D., Hamann T.W. (2016). Charge distribution in nanostructured TiO_2_ photoanode determined by quantitative analysis of the band edge unpinning. ACS Appl. Mater. Interfaces.

[42] Jiang X., Kuklin A.V., Baev A., Ge Y., Ågren H., Zhang H., Prasad P.N. (2020). Two-dimensional MXenes: From morphological to optical, electric, and magnetic properties and applications. Phys. Rep..

[43] Yang Q., Zhang F., Zhang N., Zhang H. (2019). Few-layer MXene Ti_3_C_2_T_x_ (T = F, O, or OH) saturable absorber for visible bulk laser. Opt. Mater. Express.

[44] Schultz T., Frey N.C., Hantanasirisakul K., Park S., May S.J., Shenoy V.B., Gogotsi Y., Koch N. (2019). Surface termination dependent work function and electronic properties of Ti_3_C_2_T_x_ MXene. Chem. Mater..

