# Supplementary material for: Effects of Ti3C2Tx MXene Addition to a Co Complex/Ionic Liquid-Based Electrolyte on the Photovoltaic Performance of Solar Cells
Source: Molecules. 2024 Mar 18;29(6):1340. doi: 10.3390/molecules29061340 (PMC10974105; doi:10.3390/molecules29061340)
Supplement: Supplementary file 1 [file molecules-29-01340-s001.zip › molecules-2899769-supplementary.pdf]

Article (Electronic Supplementary Materials)

Effects of Ti_3C_2Tx MXene addition to a Co complex/ionic liquid-based electrolyte on the photovoltaic performance of solar cells

Ju Hee Gu, Dongho Park, Kyung-Hye Jung, Byung Chul Lee† and Yoon Soo Han*

Department of Advanced Materials and Chemical Engineering, Daegu Catholic University, Gyeongbuk 38430, Republic of Korea; gjh3170@naver.com (J.H.K.); donggeulhonyang@naver.com (D.P.); khjung@cu.ac.kr (K.-H.J.); bclee@kbsc.ac.kr (B.-C.L.)

* Correspondence: yshancu@cu.ac.kr

† Current address: Kyongbuk Science College, Chilgokgun, Kyongsangbukdo 39913, Republic of Korea

1. Photovoltaic performance of DSCs with or without $Ti_3C_2T_x$ MXene

Table S1. Photovoltaic parameters of I- and Co-DSCs with or without $Ti_3C_2T_x$ MXene

Solar cells	J_{sc} (mA/cm²)	V_{oc} (V)	*FF* (%)	*PCE* (%)
I-DSC (without $Ti_3C_2T_x$)	20.45	0.652	56.51	7.54
	17.86	0.682	58.73	7.15
	18.98	0.676	60.69	7.78
	18.08	0.696	54.87	6.91
	18.84 ± 1.18	0.677 ± 0.018	57.70 ± 2.55	7.35 ± 0.39
I-DSC (with $Ti_3C_2T_x$)	20.92	0.636	54.71	7.28
	20.17	0.654	60.27	7.95
	19.58	0.654	58.58	7.50
	19.12	0.658	55.6	7.00
	19.95 ± 0.78	0.651 ± 0.010	57.29 ± 2.59	7.43 ± 0.40
Co-DSC (without $Ti_3C_2T_x$)	16.82	0.730	57.18	7.02
	14.41	0.780	64.66	7.27
	15.65	0.776	59.26	7.20
	14.96	0.752	64.23	7.23
	15.46 ± 1.04	0.760 ± 0.023	61.33 ± 3.70	7.17 ± 0.11
Co-DSC (with $Ti_3C_2T_x$)	18.05	0.726	64.04	8.39
	19.04	0.750	59.96	8.56
	18.45	0.760	64.23	9.01
	16.83	0.748	66.42	8.36
	18.09 ± 0.94	0.746 ± 0.014	63.66 ± 2.69	8.58 ± 0.30

2. V_{oc} variations of I-DSCs with exposure time

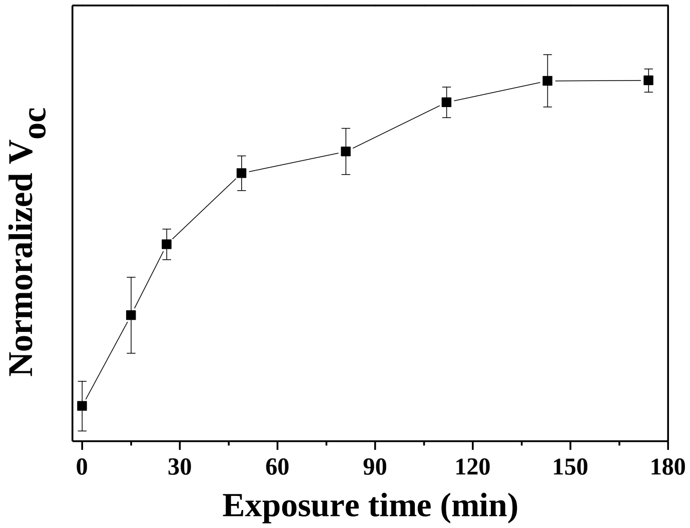

Figure S1. Improvement in V_{oc} values by exposing I-DSCs to AM 1.5 light.

3. UV-visible absorption spectra of redox mediators and dye

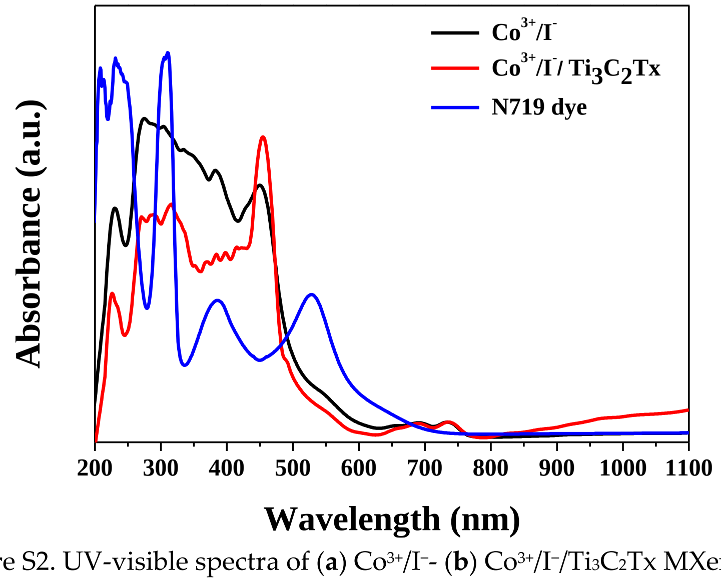

Figure S2. UV-visible spectra of (**a**) Co^{3+}/I^-- (**b**) $Co^{3+}/I^-/Ti_3C_2Tx$ MXene-based redox mediators, and (**c**) N719 dye.

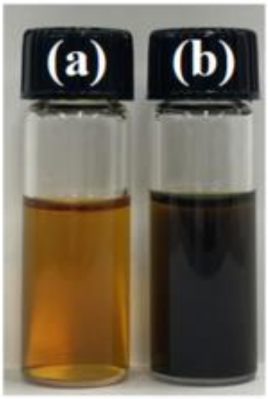

Figure S3. Photographs of (**a**) Co^{3+}/I^{-}- (**b**) $Co^{3+}/I^{-}/Ti_3C_2Tx$ MXene-based redox mediators.

4. Materials for fabrication of DSCs

Commercial goods such as transparent glass substrate coated with fluorine-doped tin oxide with a sheet resistance of ~7 Ω/square (FTO glass) (TCO22–7), TiO_2 paste for the mesoporous layer (Ti–nanoxide T/SP), TiO_2 paste for the scattering layer (Ti–nanoxide R/SP), N719 dye (Ruthenizer 535–bisTBA), and hot-melt adhesive (Metlonix 1170–25, DuPont Surlyn) were all purchased from Solaronix (Aubonne, Switzerland). Titanium diisopropoxide bis(acetylacetonate) (TPA), $TiCl_4$, FK209, MPII, bis(trifluoro-methane)sulfonimide lithium salt (LiTFSI), and TBP were purchased from Sigma-Aldrich (St. Louis, MO, USA). Platinum paste (PT–1) produced by Dyesol-Timo JV (Seoul, Korea) was selected as the source of the Pt counter electrode. The acetonitrile solvent used to prepare the liquid electrolytes was procured from Daejung Chemicals and Metals Co., Ltd. (Gyeonggi-do, Korea). Single-layer Ti_3C_2 colloid in acetonitrile (2 mg Ti_3C_2/mL) (BK2020082105-08) was purchased from Beijing Beike New Material Technology Co., Ltd. (Jiangsu, China). All the chemicals used for DSC fabrication were used without further purification. The single-layer Ti_3C_2Tx MXene structure is illustrated in below Figure S4. The chemical structures of the main components (FK209 and MPII) and additives (LiTFSI and TBP) of the electrolyte are shown in Figure S5.

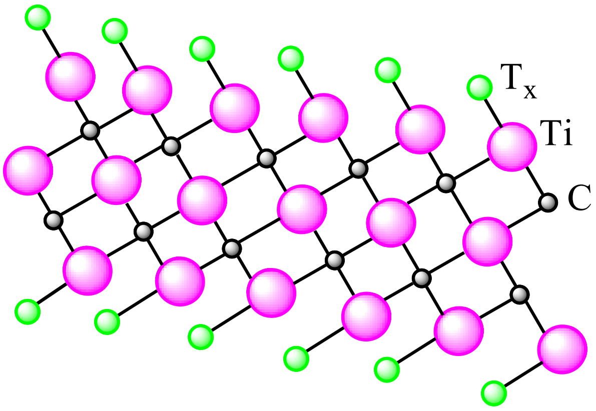

Figure S4. Illustration of single-layered Ti_3C_2Tx MXene structure.

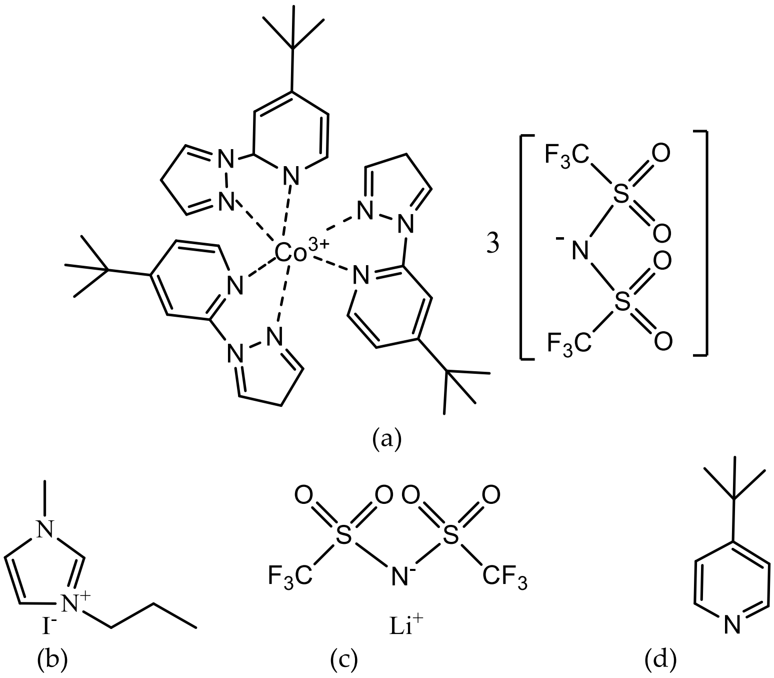

Figure S5. Chemical structures of (**a**) FK209, (**b**) MPII, (**c**) LiTFSI, and (**d**) TBP.

5. Detailed fabrication conditions of working and counter electrodes for DSCs

To prepare the working electrodes, the FTO glasses were cleaned in a detergent solution with sonication for 20 min, and then thoroughly rinsed with deionized (DI) water and ethanol. The ethanolic solution of TPA (0.3 M) was spin-coated on a cleaned FTO glass and dried for 2 min at 150 °C. The TiO_2 pastes used for the mesoporous and scattering layers were sequentially coated on the TPA-coated substrate using the doctor blade method, which was followed by sintering at 500 °C for 60 min. The sintered TiO_2 layers were then soaked in a mixture of water and $TiCl_4$ (40 mM) for 30 min. After rinsing with water and ethanol, the layers were annealed at 500 °C for 60 min. The resulting TiO_2 photoanodes (glass/FTO/TiO_2) were immersed in 0.5 mM of ethanolic N719 dye solution for 8 h to obtain the working electrodes.

To prepare the counter electrodes, two holes were formed in the FTO glasses using a drill, and the glasses were cleaned using the method described above. Pt layers were formed on the FTO glass via the doctor blade coating method using Pt paste, followed by calcination at 400 °C for 30 min.

A 25-μm-thick hot-melt adhesive was placed between the working and counter electrodes and then annealed for 10 min at 120 °C to seal the two electrodes. The Co^{3+}/I^{-}-based liquid electrolytes with or without $Ti_3C_2T_x$ MXene were injected into the cells through one of the two small holes predrilled into the counter electrodes. By sealing the two holes, we were able to fabricate DSCs with a 25 mm^2 active area.

6. Instrumental measurements

The photocurrent voltage measurements, electrochemical impedance spectroscopic (EIS) analyses, dark current studies and OCVD measurements were all performed using a CompactStat potentiostat (Ivium Technologies BV; Eindhoven, The Netherlands). A PEC-L01 solar simulator system equipped with a 150 W xenon arc lamp (Peccell Technologies, Inc.; Yokohama, Japan) was used as the light source. The light intensity was adjusted to 1 sun (100 mW/cm^2) using a silicon photodiode (model PEC-SI01, Peccell Technologies, Inc.; Yokohama, Japan). The UV–vis absorption spectra were obtained using a

SINCO NEOSYS–2000 spectrophotometer (Seoul, Korea). The active areas of the dye-adsorbed TiO_2 films were estimated using a digital microscope camera (SZ61, Olympus Corporation, Tokyo, Japan) and image analysis software.
